# Genome-Wide Gene Expression Analysis in Response to Organophosphorus Pesticide Chlorpyrifos and Diazinon in *C. elegans*

**DOI:** 10.1371/journal.pone.0012145

**Published:** 2010-08-16

**Authors:** Ana Viñuela, L. Basten Snoek, Joost A. G. Riksen, Jan E. Kammenga

**Affiliations:** Laboratory of Nematology, Wageningen University, Wageningen, The Netherlands; Cinvestav, Mexico

## Abstract

Organophosphorus pesticides (OPs) were originally designed to affect the nervous system by inhibiting the enzyme acetylcholinesterase, an important regulator of the neurotransmitter acetylcholine. Over the past years evidence is mounting that these compounds affect many other processes. Little is known, however, about gene expression responses against OPs in the nematode *Caenorhabditis elegans*. This is surprising because *C. elegans* is extensively used as a model species in toxicity studies. To address this question we performed a microarray study in *C. elegans* which was exposed for 72 hrs to two widely used Ops, chlorpyrifos and diazinon, and a low dose mixture of these two compounds. Our analysis revealed transcriptional responses related to detoxification, stress, innate immunity, and transport and metabolism of lipids in all treatments. We found that for both compounds as well as in the mixture, these processes were regulated by different gene transcripts. Our results illustrate intense, and unexpected crosstalk between gene pathways in response to chlorpyrifos and diazinon in *C. elegans*.

## Introduction

Organophosphorus pesticides (OPs) are widely used to control agricultural and household pests, and consequently these compounds have been well studied for their impact on gene function and physiology. Initially, OPs were designed to affect the nervous system of pest organisms by inhibiting acetylcholinesterase (AChE). This leads to a cholinergic hyper stimulation as a common mode of action [1]. However, mounting evidence suggests that this is not the only process that is being affected. For instance, mice lacking AChE are hypersensitive to OPs toxic effects, indicating that OPs inhibit targets other than AChE [2]. In addition, neurotransmitter receptors, proteases and several other enzymes interact with OPs to modify the consequences of AChE inhibition [3]. Moreover, many OPs alter immune functions in mammals by oxidative damage, metabolism modifications and stress-related immunesupression [4].

Of the hundreds of OPs, chlorpyrifos (CPF) and diazinon (DZN) are intensively used as insecticides and acaricides. CPF is the most thoroughly studied OP and, like DZN and most OPs, is desulfurated by cytochrome P450 enzymes (CYPs). CPF and DZN share detoxification pathways and molecular targets as they are structurally similar [5]. DZN, however, induces a different inhibition ratio of AChE [6]. Some studies also investigated the response of AChE to a combination of CPF and DZN [7], [8]. Their results suggest a greater inhibition of cholinesterase by CPF than by DZN and a greater influence of CPF in the combination. Likewise, kinetic models of humans and rats exposed to CPF and DZN indicate an interaction between both compounds at the enzymatic level. In particular, CPF and DZN seem to compete for common CYPs [9].

Despite our knowledge about OPs responses in different organisms, little is known about genome-wide gene expression responses of OPs in the nematode *Caenorhabditis elegans*. This is surprising because *C. elegans* has been well established as a model for understanding human toxicology, especially for studying neurotoxic compounds like OPs [10]. The effects of CPF on *C. elegans* have also been investigated in contrast to other chemical effects [11]. These studies showed that neurotoxic compounds affect behaviour and movement in *C. elegans*. Additionally, gene transcription changes by CPF were investigated by Roh & Choi (2008) [12] who found that CPF regulates stress related genes. Still, a comparative analysis of the genome-wide transcriptional responses of *C. elegans* to DZN and CPF has not been conducted.

Here we measured genome wide gene transcription profiles of *C. elegans* exposed to CPF and DZN and to a low dose mixture (LDM) of both compounds. Gene Ontology (GO) and domain enrichment analysis illustrates the complexity with novel and known pathways associated to OPs response.

## Materials and Methods

### *C. elegans* culturing

The Bristol N2 strain was cultured on standard nematode growth medium (NGM) with E. coli OP50 as food source. Nematodes were bleached (0.5 M NaOH, 1% hypochlorite) to collect eggs which were inoculated in 9 cm dishes for toxicity experiments. After 72 hours, nematodes were collected in the L3-L4 stage, frozen in liquid nitrogen and kept at −80C until the RNA extraction procedure.

### Toxicant exposures

We analyzed gene expression in response to the toxicants at concentrations below the EC_50_ values for different fitness traits as reproduction (CPF: EC50 = 3.5 mg/L [13] DZN: EC_50_ = 30 mg/L [14]) or growth (CPF: EC_50_ = 14 mg/L [15]), Nematodes were exposed to 0.5 mg/L of CPF (Cyren®/Nufos®, Cheminova A/S [Lemvig, Denmark]) and 1 mg/l of DZN (Supelco [Bellefonte, Pennsylvania 16823, USA]). The low dose mixture (LDM) of the two OPs contained the sum of both single concentrations (DZN [1 mg/l] and CPF [0.5 mg/l]).

The experiment started with eggs placed on NGM dishes with the OP-treatments and *E. coli* OP50 as food source. After 72 hours, worms from 4 petri dishes were collected as one sample. A total of 6 replicates per treatment were collected (24 petri dishes), and immediately frozen in liquid nitrogen until RNA extraction. All the OPs were dissolved in acetone and added to 10 ml of NGM poured in each 9 cm petri dish used for the culture. Nematodes without treatments were grown simultaneously with the same concentrations of acetone in a control culture.

### Microarray experiments

RNA from nematodes was extracted following the Trizol method, and the RNeasy Micro kit (Qiagen, Valencia, CA, USA) was used to clean up the samples. Labeled cDNA was produced with the kit Array 900 HS from Genisphere and Superscript II from Invitrogen. The 60-mers arrays were purchased from Washington University (http://genomeold.wustl.edu) and they were hybridized following the Genisphere Array 900 HS protocol with modifications. Extracts from CPF, DZN and the CPF/DZN combination exposures were hybridized with the control samples in each array. Six independent biological replicates were used per treatment to produce six replicate microarrays per experiment in a dye-swap design.

### Microarray Analysis

A Perking & Elmer scanner was used to extract the raw intensities from the microarrays. Normalization within arrays and normalization between arrays of raw intensities was done using loess method [16] and aquantile method [17], respectively. Both methods are included in the Limma package [18] from R software (www.r-project.org/). The Rank Product package [19] was used to identify the differentially expressed genes between controls and treatment in each experiment. Briefly, genes were ranked based on up- or downregulation by the treatment in each experiment. Then, for each gene a combined probability was calculated as a rank product (RP). The RP values were used to rank the genes based on how likely it was to observe them by chance at that particular position on the list of differentially expressed genes. The RP can be interpreted as a p-value. To determine significance levels, the RP method uses a permutation-based estimation procedure to transform the p-value into an e-value that addresses the multiple testing problem derived from testing many genes simultaneously. Genes with a percentage of false-positives (PFP) <0.05 were considered differentially expressed between treatments and control in each experiment.This method has the advantage to identify genes with a response to the toxicants even when the absolute effect of the response was low. Because we used sub-lethal concentrations of the toxicants, methods that use thresholds based on absolute fold change would not identify small changes in gene expression. Moreover, RP has proved to be a robust method for comparing microarray data from different sources and experiments [20].

Gene Ontology (GO) data and functional domain data were extracted from Wormbase release WB195 using the R package BioMart [21]. GO terms and domains with less than 4 genes were discarded. Over-represented groups of GO terms and functional domains were identified using a hypergeometric test, with a threshold of p-value<0.01. The hypergeometric test compared a group of 396 GO terms, with 16,947 annotated genes, with the GO terms associated with the significantly regulated genes in each treatment (551,245 and 233 for CPF, DZN and the LDM). For functional domains analysis, 1003 InterPro and Pfam domain terms were used, with 8682 annotated genes.

The same hypergeometric test was used to determine significant regulation of the different pathways analyzed. The lists of regulated genes, in this case, were extracted from the original publications. Groups sizes were 541 genes for the daf-16 pathway, 255 for the etl-2 pathway, 305 for the mdt-5 pathway, 320 for the dbl-1 pathway, 143 for the pmk-1 pathway, and 290 genes for Cd responsive genes. Annotated genes were all the unique genes on the microarray, 18893.

Microarray data have been deposited in Gene Expression Omnibus (www.ncbi.nlm.nih.gov/geo/), accession number GSE16719.

## Results and Discussion

### Effects of CPF and DZN on transcript levels in *C. elegans*

**Figure 1:**
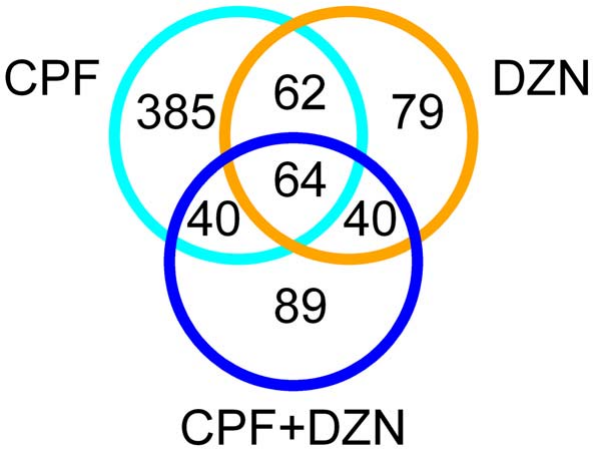
Transcriptional response to CPF, DZN and combination. Venn diagram showing significantly regulated genes by CPF (cyan circle), DZN (orange circle), a combination of both (CPF + DZN, blue circle) and their overlap.

We analyzed global gene transcription profiles of C. elegans treated with CPF or DZN. These profiles were used to reveal transcriptional responses of each OP. Compared to control worms, 551 and 245 genes were significantly regulated in CPF and DZN treated worms, respectively. Of these genes, 126 were regulated by either compound (Figure 1 and Table S1).

**Figure 2:**
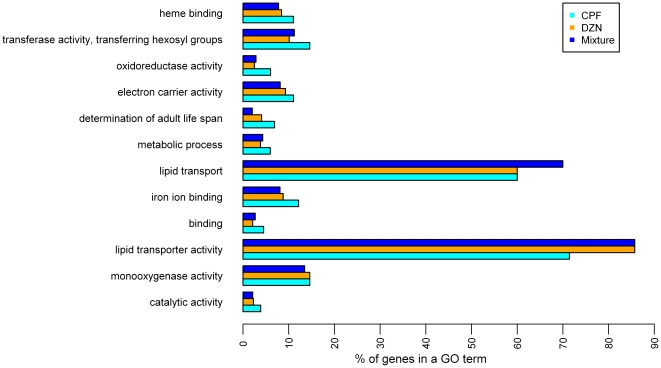
Differentially expressed genes within enriched GO terms in CPF, DZN and combination. Percentage of genes within each GO terms significantly regulated in each treatment: CPF (cyan circle), DZN (orange circle) and a combination of both (blue circle). Only significantly enriched GO terms for all the treatments are shown (*p-value*<0.01 using hypergeometric test). Full list of GO terms can be found in Table S2.

**Figure 3:**
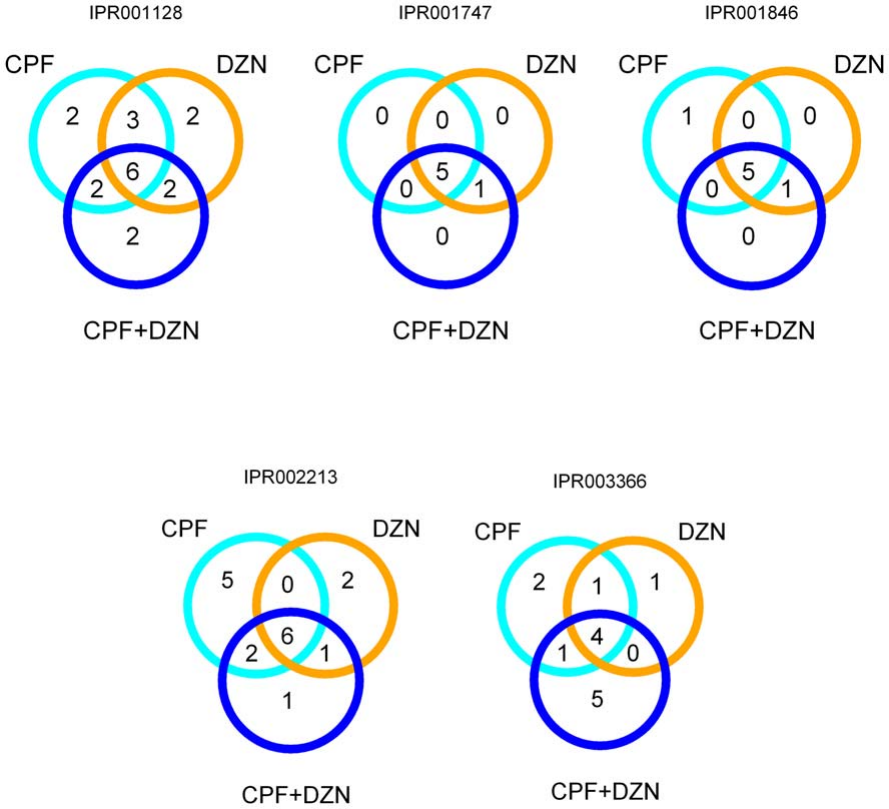
Differentially expressed genes within enriched domains in CPF, DZN and combination. Major functional groups represented in all the treatments: CPF (cyan circle), DZN (orange circle) and a combination of both (blue circle). The number of genes that show a functional domain are shown in each case, indicating the common regulated genes. Only functional domains common and significant (*p-value*<0.01, hypergeometric test) within the three treatments are shown. Functional domain categories were extracted from defined proteins domains in Wormbase release 190 using BioMart. Domains description: IPR001128 = Cytochrome P450; IPR001747 = Lipid transport protein, N-terminal; IPR001846 = von Willebrand factor, type D; IPR002213 = UDP-glucuronosyl/UDP-glucosyltransferase; IPR003366 = CUB-like region.

To gain insight into the biological processes associated with the regulated genes, we determined which GO annotation terms were over-represented. In both treatments, significantly enriched GO terms (p<0.01, hypergeometric test) were related with detoxification (monooxygenase activity), metabolic process, and transport of lipids (Figure 2, Table 1 and Table S2). To add meaningful information to the results from the GO term enrichment we extended our investigation by using a similar analysis with protein domains associated to the regulated genes as categories (Figure 3 and Table S2). The significantly overrepresented groups (p<0.01, hypergeometric test) also included domains related to detoxification, stress-response, and transport of fatty acids. In addition, we also found significantly enriched domains associated with other metabolic pathways and immune response.

**Table 1:** Major Gene Ontology (GO) terms represented in the different treatments.

Definition	GO terms	Genes in CPF	Genes in DZN	Genes in mixture	Common genes in all three treatments
catalytic activity	GO:0003824	22	13	12	2
monooxygenase activity	GO:0004497	13	13	12	6
lipid transporter activity	GO:0005319	5	6	6	5
binding	GO:0005488	32	15	19	4
iron ion binding	GO:0005506	18	13	12	6
lipid transport	GO:0006869	6	6	7	5
metabolic process	GO:0008152	36	23	26	8
Determination of adult life span	GO:0008340	17	10	5	3
electron carrier activity	GO:0009055	19	16	14	6
oxidoreductase activity	GO:0016491	17	7	8	1
transferase activity, transferring hexosyl groups	GO:0016758	13	9	10	6
Heme binding	GO:0020037	17	13	12	6

A summary of common significant (p-value<0.01, hypergeometric test) GO terms in each treatments is shown. The final column show the number of common genes within a GO term in all the three treatments. Only GO terms significant in all the three treatments are shown. A complete list with all the GO terms and its correspondent p-values can be found in Table S1.

### Detoxification of CPF and DZN

Enzymes and functional domains associated with detoxification in C. elegans are mainly cytochrome P450 (CYP) and short-chain dehydrogenase (SDR) in phase I of the xenobiotic metabolism, and UDP-glucuronosil/transferase (UGT) and glutathione S transferase (GST) in phase II [22]. Their implication in the detoxification of CPF and DZN has been well characterized in different organisms [7], [23]. In our experiment, 19 genes that code for domains related to phase I responded to the CPF treatment, whereas 14 genes with these domains responded to the DZN treatment. Of those genes, 10 were shared between CPF and DZN treatment, of which nine were CYPs. Five of those shared CYPs belong to the cyp-35 subfamily, which has previously been identified as strongly inducible by a range of xenobiotics in C. elegans [24]. In addition, only one gene (F25D1.5) coding for an SDR domain was regulated in both treatments.

Also genes coding for proteins with domains involved in phase II of detoxification were affected by CPF (21) and DZN (14). From those genes, 8 were affected by both treatments. One of these common genes was cdr-1 (F35E8.1), a member of the cadmium responsive genes family. Two other genes from this family were affected only by CPF, cdr-4 (K01D12.11) and cdr-6 (K01D12.12), and cdr-5 (K01D12.14) only by DZN. Whereas cadmium modifies the expression levels of cdr-4 and cdr-1, the level of expression of cdr-5 and cdr-6 are impervious to this metal's presence [25]. Moreover, other stressors are capable of modulating cdr-4 expression, but until now, cdr-1 transcription has only been induced by cadmium.

### Role of the daf-16 pathway in response to OPs

Another gene involved in phase II of detoxification and shared between treatments was gst-10 (Y45G12C.2). This gene acts downstream of daf-16 (R13H8.1), a FOXO class transcription factor involved in the insulin-like growth factor signalling in C. elegans [26]. Detoxification genes are known to constitute a big group among the identified up-regulated genes by daf-16 [26], [27]. Other genes acting downstream daf-16 have also been associated with toxic stress response. We found from those groups metallothioneins (mtl) and vitellogenins (vit) to be affected by CPF or DZN. DZN decreased the transcript levels of mtl-2 (T08G5.10), while CPF did not regulate any of the two mtl genes present in C. elegans. Furthermore, vitellogenin genes, lipid-binding proteins involved in lipid mobilization, were down-regulated in both treatments. CPF modified the expression of 5 out of 6 members of the vit gene family (vit-1 to vit-3, vit-5, and vit-6) while DZN regulated all 6 vit genes. Furthermore, both OPs regulated collagen genes (34 by CPF, 16 by DZN) and major sperm proteins type genes (33 by CPF, 3 by DZN) acting downstream of daf-16. Many of these, have been associated with stress responses, although their specific connection to these responses remains unresolved [28], [29]. The overlap in genes affected by CPF and/or DZN and the daf-16 pathway suggest that this pathway is a primary response pathway following exposure to OPs.

### OPs affect innate immunity genes

The insulin-like signalling pathway regulated by daf-16 is known to induce the immune response as part of a general stress response [30]. On the other hand, Lewis et al. (2009) [31] showed that OPs regulate daf-16 in C. elegans as a key modulator of physiological responses other than stress, including innate immune response. Therefore, it is not surprising to find that lys-7 (C02A12.4), a gene coding for a well defined antimicrobial lysozyme, was regulated by both toxicants. This gene is presumed to promote resistance to infection [32]. Interestingly, while lys-7 is up-regulated under both OP treatments and suggests an immune response, spp-1 (T07C4.4) was down-regulated by CPF, but not by DZN. This saposin is also a well known pathogen responsive gene. However, suppression of spp-1 during Pseudomonas aeruginosa infection seems to be under regulation of a different pathway than daf-16 [33]. Therefore, other gene pathways may participate in parallel to daf-16 insulin-like pathway to promote stress and infection responses in C. elegans as a result of OPs exposure.

To further investigate an innate immunity response to OPs, we compared the regulated genes in our experiments with genes associated with immune response and stress in C. elegans. An indication that different genes are involved in stress and infection response was found by Shapira, et al. 2006 [34]. The authors compared genes regulated in response to P. aeruginosa with those affected by cadmium. They reported several overlapping genes among these two responses, but they concluded that a reaction to infection is largely distinct from stress response. Based on this, we considered that an innate immune reaction signature may be distinguishable from OP stress response in C. elegans. Subsequently, we also compared our set of genes with the same set of cadmium responsive genes and pathogens responsive genes Shapira et al. used (Table 2). We found more genes overlapping between the OPs affected genes and the cadmium responsive genes. The similarities between genes modulated by OPs and by cadmium suggest the implication of a common response mechanism of gene expression responses to toxic compounds. Nevertheless, the larger number of genes affected by OPs as well as P. aeruginosa infection supports the idea of an innate immune response to OPs. For example, a CUB-like domain (also known as DUF141) containing gene (F55G11.2) is induced during infection but repressed under cadmium exposure. This gene was up-regulated in both OP treatments. Another gene (F08G5.6) up-regulated in all OP treatments is strongly induced by infection but not by cadmium.

**Table 2:** Pathways involved in OPs response.

Pathway/mechanism	num. genes	CPF		DZN		CPF + DZN		Reference
		Overlap. genes	p-value	Overlap. genes	p-value	Overlap. genes	p-value	
DAF-16	541	47	1.679e-10	36	2.920e-16	29	3.242e-11	[26]
ELT-2	255	30	1.779e-10	18	1.723e-09	21	3.562e-12	[34]
MDT-15	305	57	1.346e-28	29	1.319e-17	29	8.589e-18	[41]
DBL-1/SMA-9	320	31	1.224e-08	15	7.045e-06	22	2.829e-11	[40]
PMK-1	143	13	0.00019	5	0.01267	5	0.01190	[36]
Cd	290	44	9.917e-19	44	8.403e-35	40	2.447e-30	[28]

Pathway/mechanism investigated were: genes downstream *daf-16* (DAF-16), genes regulated by the GATA transcription factor ELT-2 (ELT-2), genes regulated by the Mediator subunit MDT-15 (MDT-15), genes downstream *pmk-1* (PMK-1) and cadmium responsive genes (Cd). Number of genes associated with each pathway or mechanism are shown. Overlapping genes are the number of genes belonging to a pathway that are regulated in each treatment. P-values were calculated using hypergeometric test.

**Figure 4:**
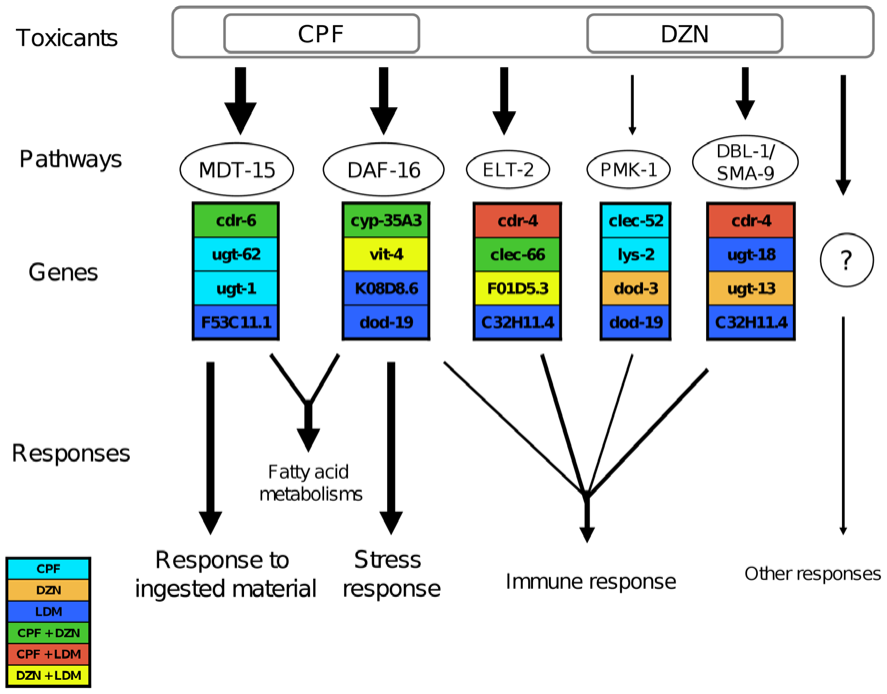
A model for the transcriptional responses to CPF and DZN in *C. elegans.* Exposure to the toxicants CPF, DZN or a low dose mixture (LDM) of both organophosphates modulates the expression of genes in common pathways. The most regulated pathways are shown with the thicker arrows: genes regulated by MDT-15 (MDT-15), genes downstream daf-16 (DAF-16), and genes downstream elt-2 (ELT-2). For details, see Table 2. Despite this common regulated pathways, the three treatments regulated different genes to induce common responses such a stress response, response to ingested material or immune response. Here we show some regulated genes by chlorpyrifos (CPF), diazinon (DZN) and a low dose mixture (LDM). With different colors are shown common regulated genes by chlorpyrifos and diazinon (CPF + DZN), by chlorpyrifos and low dose mixture (CPF + LDM), and by diazinon and low dose mixture (DZN + LDM). An intense crosstalk between gene pathways in response to the toxicants may be explain by the genes shared between pathways. For example, *cdr-4* was regulated by CPF and LDM but not by DZN. Previous studies showed that its expression is modulated by ELT-2 and DBL-1, two transcription factors implicated in responses to infection. Exposure to the toxicants modulated the expression of many genes from those pathways that, acting in parallel, induced the detected responses. We analyzed regulated genes within the Different genes within those common pathways regulate the common responses to all treatments.

The same study from Shapira and colleagues identified ELT-2, a GATA transcription factor, as key regulator for transcriptional responses to infection. ELT-2 together with SKN-1, also a transcription factor, have been proposed as complementary regulators of spp-1 during infection [33]. Since spp-1 is also regulated by the daf-16 pathway, genes acting downstream of elt-2 and skn-1 may act in parallel to induce a combined stress-immune response to CPF and DZN (Figure 4). In that sense, SKN-1 is known to mediate an expression response of genes involved in phase II of detoxification. To induce these genes, SKN-1 is phosphorylated through the PMK-1 p38 mitogen-activated protein kinase pathway (p38 MAPK) [35]. Genes regulated by pmk-1 are essential for immune and stress responses in C. elegans. These genes code for antimicrobials proteins with CUB-like domains, C-type lectins domains, ShK domains, and DUF274 domains [36]. Our domain enrichment analysis showed that OPs regulated genes involved in phase II of detoxification. It also showed an overrepresentation of domains associated to innate immune response through the pmk-1/skn-1 pathway (Table S2). Therefore, we compared our data sets with the genes downstream pmk-1 identified by Troemel, et al. (2006) [36] (Table 2). We found significant overlapping between genes belonging to this pathway and the genes that were regulated in each treatment (13 genes, p-value<0.01) for CPF response, but not for DZN (5 genes, p-value = 0.012). This strengthens the implication of this pathway in response to CPF and indicates differences in immunomodulation between CPF and DZN. Those differences have been already reported in in vitro studies with human cells, where CPF showed a stronger effect in cell viability and some immune parameters [37]. Altogether, CPF and DZN modified innate immunity related genes in C. elegans in a different manner and as a part of complex stress response.

### Transcriptional effects of a the low dose mixture (LDM)

In the LDM, 233 genes were significantly regulated (Table S1), of which 64 were affected by all, the combination and the both single compound treatments (Figure 1). More genes (89) were affected by the LDM only. The GO terms enrichment analysis (Figure 2, Table 1 and Table S2) showed a significant modulation of detoxification pathways, stress response, and transport and metabolism of lipids, which is highly similar to the single compound treatments. Moreover, the domain enrichment analysis identified differentially expressed genes that code for CYP, SDR, GST, UDP, C-type lectin, and CUB-like domains (Figure 3 and Table S2).

Regulation of CYPs genes is required for detoxification of OPs in the single and combined treatments. Our findings showed that the OP combination regulated 12 CYPs (IPR001128), 6 of which were also affected by CPF and DZN in single treatments. On the other hand, 2 CYPs were regulated only in the combined treatment. Differences in the regulated CYPs were expected, because OPs seem to compete for binding sites of CYPs [9]. Moreover, in vitro analysis of CYPs metabolism of CPF and DZN indicates that CPF inhibits the metabolism of DZN to IMHP (the inactive pyrimidol derived from DZN), with no effect of DZN over CPF metabolism. This inhibition seems to promote oxon synthesis from DZN, and therefore, increases the total levels of oxon in the organism after a treatment of more than one compound. Moreover, the toxicity of OPs is determined by a balance between bioactivation (production of oxon metabolite) and detoxification (inactivation of OPs and its intermediate oxon) [5]. Therefore, higher levels of DZN bioactivation, due to modification of this balance, may be a reason for a greater-than-additive inhibition of cholinesterase enzymes in mixtures of OPs [14], [38]. At the transcriptional level however, the number of CYPs regulated by the combined treatment do not suggest a higher bioactivation. Even though the combined treatment contained a higher concentration of toxicants, a similar number of genes involved in detoxification was regulated in the combined and the single treatments. These results were not contradictory to the previous studies, since a direct comparison between transcriptional and physiological studies is difficult. Nevertheless, our results indicated that dissimilar genes were involved in the regulation of detoxification of the different OPs treatments and may explain the differences between the LDM and the single treatments.

Other processes were modulated by all treatments besides detoxification, these include: stress, fat metabolism and innate immunity. The overlap between the analyzed pathways for the single treatments and the regulated genes in the combinations was significant (Table 2). Interestingly, as it is shown in the last column of Table 1, the number of common genes associated to some of these responses was low. For example, the modulation of stress and innate immune genes was observed for the combined treatment. Genes involved in response to infections were up-regulated such as F55G11.2, F08G5.6 and genes with the CUB-like functional domain (Figure 3 and Table S2). This again suggests an innate immune response to OPs. Furthermore, the combined treatment showed up-regulation of sma-9 (T05A10.1), a gene coding for a transcriptional factor involved in body size regulation. This transcriptional factor modulates a subset of genes in the TGF-β signalling pathway which is associated with innate immunity, aging and germ line regulation [39]. Of the identified genes in the TGF-β signalling pathway [40], the combined treatment regulated 22 genes (Table 2). A closer look to the regulated genes by CPF and DZN showed also the implication of this pathway in their transcriptional responses. Once more, as in response to the pmk-1 pathway, CPF seems to regulate more genes implicated in innate immunity, but the LDM did not show a stronger influence. Likewise, genes downstream daf-16 and elt-2 were regulated in the combined treatment.

Overall, as shown in Figure 4, the ingestion of CPF, DZN or a low dose mixture of both induced many common responses like stress and innate immunity pathways. The most regulated pathways included genes modulated by MDT-15 (MDT-15), genes downstream daf-16 (DAF-16), and genes downstream elt-2 (ELT-2). The three treatments regulated different genes within the same pathways to induce the common responses. The analysis of the regulated genes showed an intense crosstalk between gene pathways involved in the toxicants response. This suggest that the structural differences between CPF and DZN induced the expression of different genes. These differences, however, are not large enough to induce a specific different response to the exposure of CPF or DZN.

### Conclusion

We present an analysis and comparison of whole genome transcription analysis of a model organism exposed to two toxicants and a combination of both. Our results revealed that, CPF and DZN induced dissimilar genes, even though they have a similar chemical structure. The toxicants induced genes related to detoxification, stress, innate immunity and response to ingested material in single and combined treatment. The differences between transcript responses in the combined treatment suggest that the effect of a mix of low doses of CPF and DZN is not a summed effect of the single components. But at the same time, the similarities in the induced pathways (e.g.: *daf-16*, *elt-2*, *snk-1/pmk-1*, *sma-9*) indicate the regulation of similar responses to them.

## Supporting Information

Table S1 Differentially expressed genes in nematodes treated with CPF (0.5 mg/l), DZN (1 mg/l), CPF (0.5 mg/l)+DZN (1 mg/l). RankProduct (RP) was used to identify regulated genes in each of the treatments. Each worksheet contains the output for RP in one experiment. Sequence names for each regulated gene are in gene.names column. Gene.index are the microarray index for the original data. RP/sum are the rank product sum calculated per each gene. FC:(class1/class2) are the expression fold change of class 1 (treatment)/class 2 (control). pfp: estimated percentage of false positive for each gene. P-value: estimated p-value for each gene.
(0.15 MB XLS)

Table S2 Significantly enriched Gene Ontology (GO) terms and functional domains. GO terms worksheets contain full GO enriched analysis. Genes in GO database are the number of genes belonging to each GO term. GO number are the GO term. Regulated genes refer to number of significantly regulated genes in each treatment that belong to a GO term. p-values were calculated using hypergeometric test, values lower that 0.01 and more than two genes in a category were considered significant. Functional domains worksheets: Genes with the domain are the number of genes that code for a specific functional domain. The number of significantly regulated genes in each treatment that code for a specific functional domain is listed in the regulated genes column. The functional domains terms were extracted from Wormbase 195 using BioMart.
(0.03 MB XLS)
